# Nuclear medicine radiomics in digestive system tumors: Concept, applications, challenges, and future perspectives

**DOI:** 10.1002/VIW.20230032

**Published:** 2023-07-28

**Authors:** Wenpeng Huang, Zihao Tao, Muhsin H. Younis, Weibo Cai, Lei Kang

**Affiliations:** 1 Department of Nuclear Medicine, Peking University First Hospital, Beijing, China; 2 Departments of Radiology and Medical Physics, University of Wisconsin-Madison, Madison, Wisconsin, USA

**Keywords:** artificial intelligence, digestive system tumors, machine learning, medical imaging, non-invasive prediction, nuclear medicine, positron emission tomography, radiomics

## Abstract

Radiomics aims to develop novel biomarkers and provide relevant deeper subvisual information about pathology, immunophenotype, and tumor microenvironment. It uses automated or semiautomated quantitative analysis of high-dimensional images to improve characterization, diagnosis, and prognosis. Recent years have seen a rapid increase in radiomics applications in nuclear medicine, leading to some promising research results in digestive system oncology, which have been driven by big data analysis and the development of artificial intelligence. Although radiomics advances one step further toward the non-invasive precision medical analysis, it is still a step away from clinical application and faces many challenges. This review article summarizes the available literature on digestive system tumors regarding radiomics in nuclear medicine. First, we describe the workflow and steps involved in radiomics analysis. Subsequently, we discuss the progress in clinical application regarding the utilization of radiomics for distinguishing between various diseases and evaluating their prognosis, and demonstrate how radiomics advances this field. Finally, we offer our viewpoint on how the field can progress by addressing the challenges facing clinical implementation.

## 1 ∣ INTRODUCTION

Nuclear medicine imaging, mainly using positron emission tomography (PET) and its corresponding PET/computed tomography (CT) or PET/magnetic resonance imaging (MRI) multimodality methods, is rapidly developing and the most advanced molecular imaging technique for non-invasive imaging of both functional metabolic and anatomical phenomena.^[1]^ With the innovation of hardware and software, quantitative PET images have achieved enhanced spatial resolutions and greater signal-to-noise ratios. However, convntional PET quantitative parameters cannot fully reflect the spatial heterogeneity of tumor metabolism or the biological characteristics of the whole tumor.^[2]^

**FIGURE 1:**
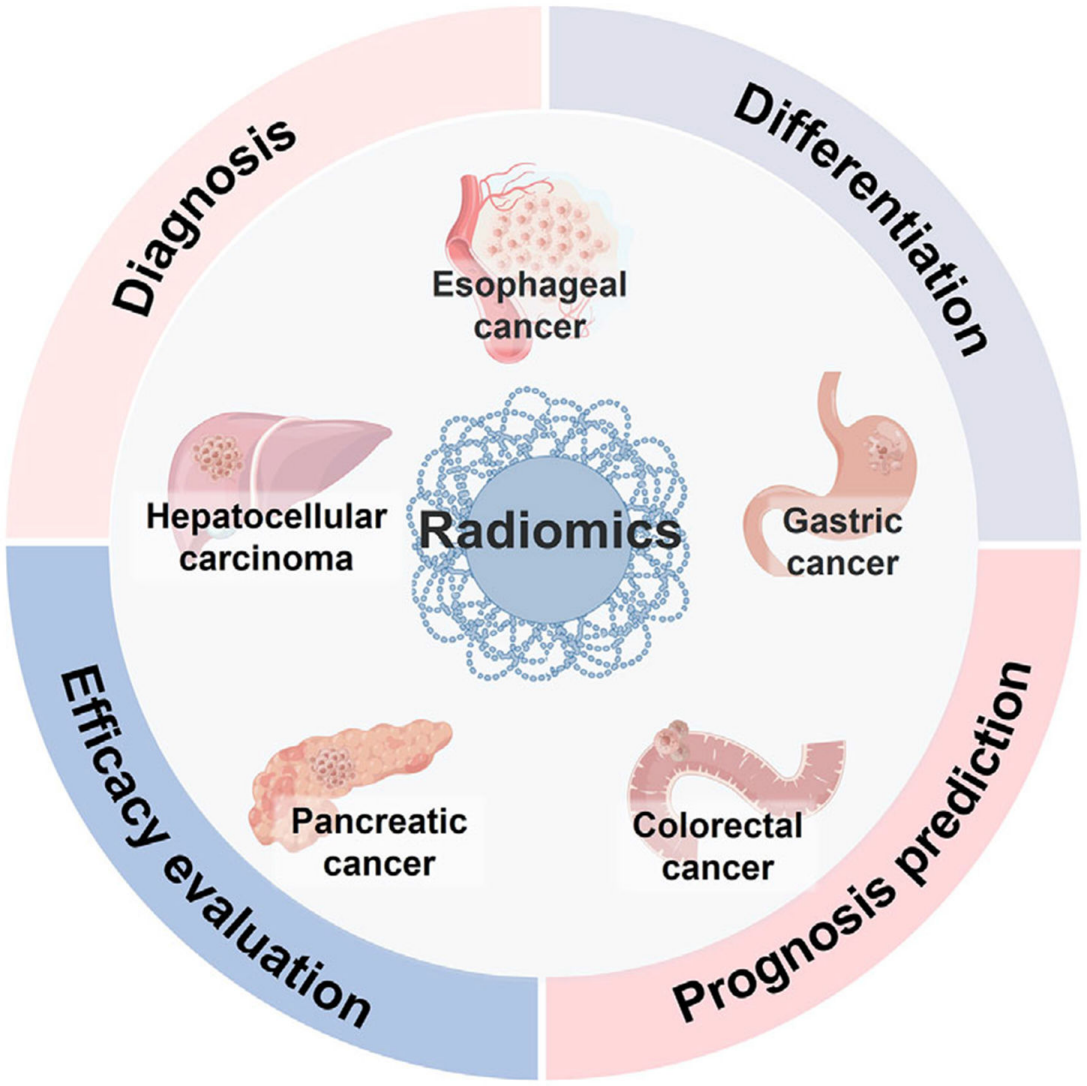
The applications of nuclear medicine radiomics in digestive system tumors.

During the “big data analysis” era, medical professionals are turning to novel technologies to interpret these large sums of data to derive diagnostic and prognostic information while ensuring personalized medicine for patients.^[3,4]^ Meanwhile, radiomics is a growing field of study that epitomizes the pursuit of precision medicine, aimed to develop novel biomarkers and provide deeper subvisual information about tissue pathology, immunophenotype, and tumor microenvironment. It uses automated or semiautomated quantitative analysis of high-dimensional images to improve characterization, diagnosis, and prognosis and has attracted increasing attention during the last decade.^[5-11]^ Radiomics is widely acknowledged to have a substantial impact on the diagnosis and treatment of tumors in the digestive system (Figure 1).

The evaluation of the role of radiomics in medical imaging has been the focus of a progressively expanding body of research since its first appearance in print by Lambin et al.^[12,13]^ According to the core collection database of the Web of Science, with the medical subject headings for “Radiomics” and “Radiomic,” the paper type “Article,” and the publication date unrestricted, 5745 papers related to imaging histology were retrieved until October 12, 2022, which shows the level of academic interest in the clinical application of imaging histology research. We summarize the reports of nuclear medicine radiomics in digestive system tumors in Table 1 (PubMed/MEDLINE and the Cochrane library database were searched from inception through February 29, 2023, using the following search terms: “Radiomics,” “Texture analysis,” “PET,” and “Nuclear medicine.” We included studies specifically focusing on digestive system tumors. PET radiomics combined with clinical data has become an effective biomarkers for tumor diagnosis, gene expression typing, preoperative evaluation of efficacy, and prognosis. Encouragingly, this approach has yielded promising results by facilitating more objective clinical decision-making.^[10,14-18]^ Although radiomics advances one step further toward the non-invasive precision medical analysis, it is still a step away from the clinical application and faces many challenges: digestive system tumors, such as those affecting the liver, pancreas, or colon, often exhibit high intra-tumoral heterogeneity. This heterogeneity can impact the reliability and generalizability of RFs. Additionally, the location and size of digestive system tumors can pose challenges for radiomic analysis. Tumors located in anatomically complex regions or near adjacent structures can be difficult to segment accurately, leading to potential errors in feature extraction. This review summarizes the available literature on digestive system tumors regarding radiomics in nuclear medicine. First, we describe the workflow and steps involved in radiomics analysis. Subsequently, we discuss the progress in the clinical application of radiomics for distinguishing between various diseases and evaluating their prognosis, demonstrating how radiomics has advanced in this field. Finally, we present our perspective on promoting the field through targeted efforts aimed at addressing specific gaps and challenges associated with clinical implementation. These include the enhancement of quality control for radiomics data, the implementation of meticulous standardization and harmonization across various modalities, the fostering of data sharing and management practices, and the utilization of advanced machinelearning (ML) algorithms and artificial intelligence techniques.

## 2 ∣ RADIOMICS WORKFLOW

**FIGURE 2:**
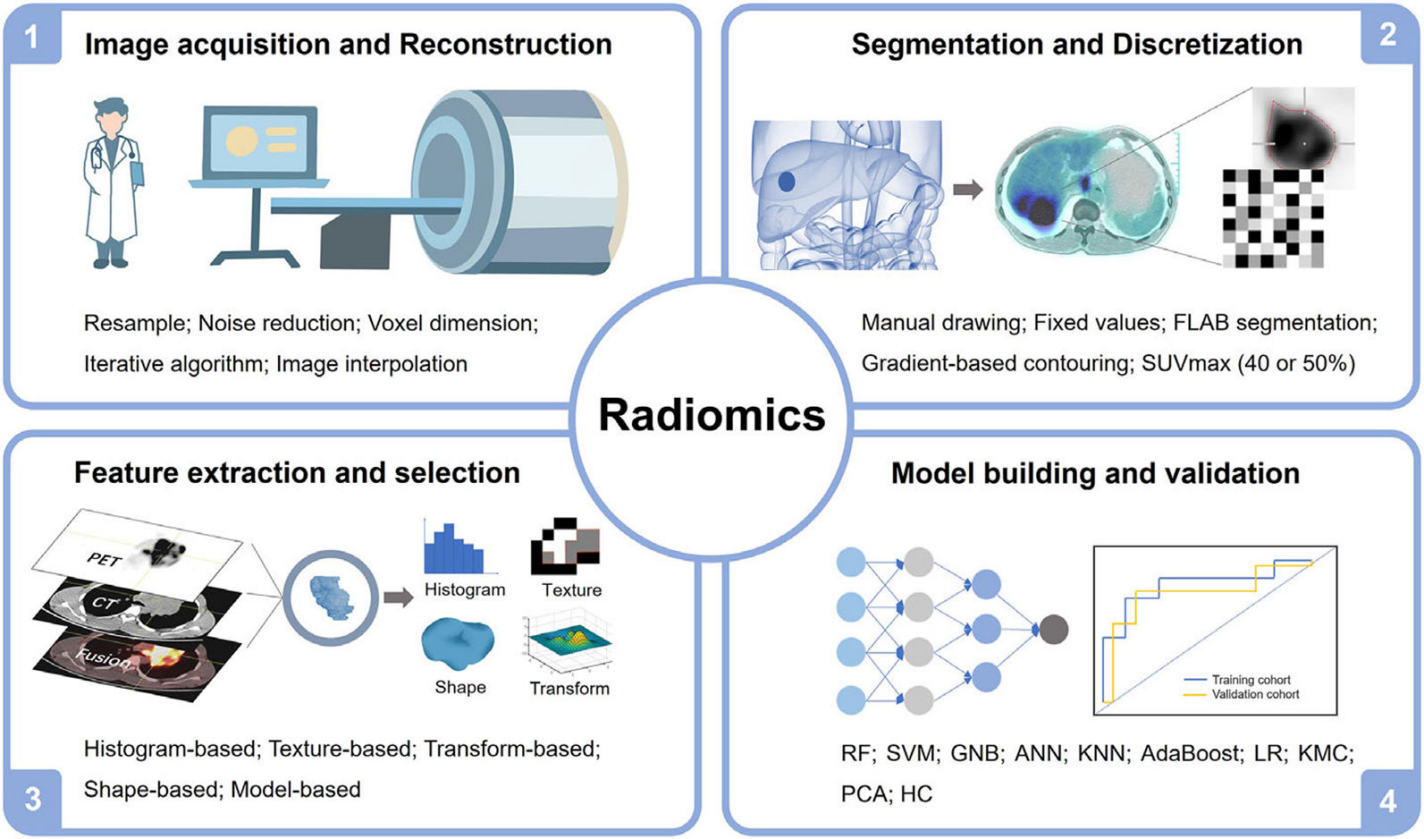
The flowchart of radiomics research analysis of nuclear medicine. AdaBoost, adaptative boosting; ANN, artificial neural network; FALB, fuzzy-locally-adaptive-Bayesian segmentation; GNB, Gaussian naive Bayes; HC, hierarchical clustering; KMC, K-means clustering; KNN, *k*-nearest neighborhood; LR, linear regression; PCA, principal component analysis; RF, random forest; SVM, support vector machine.

The workflow of radiomics research analysis of nuclear medicine in digestive system tumors is divided into four categories: acquisition and reconstruction of PET images, segmentation of tumors, extraction and identification of radiomics features (RFs), and building and validation of radiomics models (Figure 2). Each of these steps affects the outcome of radiomics analyses as well as the need to overcome unique challenges before from a concept to clinical use.^[18,41-42]^

### 2.1 ∣ Image acquisition and quality control

Image quality plays a major role in RFs. In clinical practice, PET, PET/CT, and PET/MR images are acquired using a wide range of scanning devices and manufacturers. Recent literature reviews have addressed the methods, pitfalls, and challenges associated with radiomics analysis of PET images.^[43-47]^ Variations in acquisition type (static vs. dynamic), reconstruction parameters, voxel size, and delineation appear to affect the extraction of RFs.^[45]^ Extensive investigations have been conducted on the impact of kernel filter, pixel size, slice thickness, kVp, and tube current (mA),^[48-52]^ demonstrating that reconstruction kernel filters (smooth and sharp) should not be used interchangeably.^[50]^ MR intensities are non-standardized and highly dependent on the manufacturer, sequence type, and acquisition parameters.^[53,54]^ Consequently, there exists a significant variability in intra- and inter-patient image intensities, which in turn affects RFs.^[53,55]^ These variations in acquisition and reconstruction parameters can influence the noise, contrast, and spatial resolution of medical images, thus impacting the subsequent measurement of shape, histogram, texture, and higher-order features extracted from the images. The fusion techniques and modalities employed in PET/CT and PET/MRI imaging similarly affect the quality and diagnostic performance of fused images.^[56]^

To produce generalizable results, standards need to be created for preparing the patient, acquiring the image, and reconstructing the image.^[3]^ A consensus-based reference value has been validated by the Image Biomarker Standardization Initiative in order to meet the need for standardization of RFs published recently.^[57]^ For radiomic studies to be comparable across different hospitals, clinics, and scanners, RFs should be standardized across centers.^[58]^ With its Research Ltd. (EARL) accreditation program, the European Association of Nuclear Medicine attempts to reduce measurement variability in multicenter clinical trials.^[59]^ The NU2-2012 image-quality phantom of the National Electrical Manufacturers Association serves as the basis for harmonizing basic standardized uptake value (SUV) features using SUV_max_, SUV_mean_, and SUV_peak_, and the results have been shown that PET/CT systems can be harmonized using reconstructions such as resolution modeling.^[60]^ In addition, Pfaehler et al.^[61]^ reported the impact of some factors on [^18^F]FDG PET-based RF dimensionality reduction and repeatability, including image reconstruction settings and methods, noise, underlying data, and different discretization and delineation methods. The performance of RFs is contingent upon various factors inherent to the underlying data, such as the function of tumor, contrast mechanism, tracer uptake levels, and distribution of uptake within the tumor. Therefore, the performance and reproducibility of RFs must be validated for each disease type and radiotracer. PET-based RFs are highly sensitive to the quality of images, emphasizing the importance of standardizing the reconstruction of PET images to facilitate their use as clinical imaging biomarkers.

### 2.2 ∣ Segmentation and discretization

The RFs are obtained from the region of interest (ROI) or the volume of interest (VOI) after segmentation. It is possible to segment data in several ways, but no uniform and optimal method is currently agreed upon, allowing semiautomatic or fully automatic segmentation. Some of these include manual drawing, certain percentages of SUV_max_ (40% or 50%), fixed values (SUV of 2.0, 2.5, 3.0), fuzzy-locally-adaptive-Bayesian segmentation and gradient-based contouring.^[45]^

To minimize user time investment and inter- and intrauser variability, automatic algorithms are preferred for segmentation. The simplicity and velocity of PET segmentation algorithms, however, often come at the expense of accuracy, and PET-based RFs have insufficient reliability to accurately quantify lesion heterogeneity.^[62,63]^ Before a PET segmentation method is translated into the clinic, it is necessary to demonstrate the reliability of its heterogeneity quantification. Liberini et al.^[64]^ evaluated the robustness of RFs as a function of intensity discretization settings and segmentation methods in [^68^Ga]Ga-DOTA-TOC PET/CT images of neuroendocrine tumors (NETs). As compared to [^18^F]FDG PET/CT images, [^68^Ga]Ga-DOTA-TOC images of NETs displayed a higher level of robustness to manual segmentation. When SUV_max_ thresholds are set at 40%, RFs are more stable among operators, but biological information may be lost as a result. Gray-level discretization has an impact on RF robustness, which depends on whether absolute or relative resampling is used. By implementing a semiautomatic segmentation algorithm, it is need to be resolved in the impact of different manual segmentation on the robustness, and SUV_max_ threshold segmentation leads to the loss of useful information.

### 2.3 ∣ Feature extraction and selection

Subsequently, thousands of human-defined and curated quantitative RFs could be extracted from the ROI/VOI, which can be roughly categorized as histogram, texture, transform, and shape.^[13,65-67]^ In addition, RFs can be classified into semantic features and agnostic features.^[8]^ By analyzing ROI qualitatively, semantic features are determined by human observers, including speculations, location, necrosis, vascularity, etc. Mathematical-based formula descriptions or ML-based algorithms are used to calculate agnostic features. We suggest that readers refer to Ref.[3] to understand the comprehensive definition of different RFs.

A key step in the radiomic process is feature selection. There are three classes of methods for selecting RFs: filter methods, wrapper methods, and embedded methods.^[68]^ In order to circumvent bias in the construction of models, it seems necessary to select RFs specifically and in a timely manner to obtain robust and relevant features.^[45,69-71]^ For radiomic analysis to be successful in clinical studies, reproducibility and generalizability are essential.^[72]^ Therefore, the selection process should eliminate unreliable features, features that are not informative and redundant. Further feature selection involves identifying the most relevant predictive features.^[73,74]^

Image processing steps are critical for ensuring the robustness of prognostic or predictive RFs in the radiomics pipeline.^[75]^ If small variations in resampling dimension heavily influence potential biomarkers, they will not be suitable for model development. Therefore, it is necessary to report their response to interpolation. There is currently no agreement on the optimal interpolation technique for [^18^F]FDG PET imaging. Whybra et al.^[75]^ investigated the impact of interpolation on the [^18^F]FDG PET-derived RFs for various isotropic voxel dimensions, using images of 465 esophageal cancer (EC) patients. Common 3D texture features were found to be sensitive to interpolation, and the investigators devised a correction methodology for features demonstrating potential systematic variation, which utilized surface fittings to establish a correlation between voxel dimensions and the percentage alteration in feature value.

No matter what algorithm with which the scan was interpolated, morphology features that are solely reliant on the mask shape exhibit same outcomes. When choosing linear interpolation over spline interpolation, many feature values showed wide variations, but the overall response categorizations remained unchanged. Lian et al.^[76]^ established a prediction system comprising a feature selection approach founded on the Dempster–Shafer theory, specifically designed to handle indeterminate and imprecise data. Given that the training samples in practical applications are often small and imbalanced, to enhance the dependability of the chosen feature subsets, a data balancing procedure along with a defined prior knowledge is deemed necessary. This group used the evidential *k*-nearest neighbor (KNN) classifier to select features with which to output prediction results. The prediction system has exhibited consistent favorable performance across synthetic and clinical datasets. To interpret changes in RFs over time, it is necessary to understand their intrinsic variability. A higher PET dose leads to improved image quality; however, this benefit is accompanied by a corresponding escalation in radiation exposure to both patients and hospital personnel, albeit at an expense.^[77]^ Undoubtedly, this poses a constraint on the diffusion of radiomic models and their practical utilization in clinical contexts. In order to prevent misinterpretation of the descriptive models developed based on PET images, the variability of PET-based RFs should be addressed properly.^[78]^ Extensive studies are currently underway on the acquisition of dynamic PET data, which encompasses dynamic whole-body imaging, as well as on the extraction of RFs from various imaging modalities.

In the context of multicenter subjects, Shiri et al.^[69]^ conducted a study to examine how various reconstruction parameters affect the variability and robustness of PET/CT image features. According to the findings, 47% of features displayed a coefficient of variation (COV) ≤5%, whereas 10% of the features demonstrated COV >20%. In addition, 100%, 44%, 41%, and 56% of geometry, intensity, texture, and matrix size-based features exhibited robustness, respectively. The robustness and variability of RFs in advanced reconstruction settings are feature dependent and subject to varying effects by different settings. To ensure reproducible quantification of tumors, RF reproducibility with low COV is preferred, while intermediate COV features should be interpreted with care, and features with high COV may be excluded from analysis.

Both PET/CT and PET/MR have significant implications in the field of tumor metabolism research. PET/CT has been partially replaced by hybrid systems combining PET and MRI.^[79]^ However, there exists variation in the RFs observed between PET/MR and PET/CT. This may result from different reconstruction processes, including the correction of attenuation, partial volume effects, and noise reduction.^[80,81]^ Liao et al.^[82]^ conducted a study to examine the comparability of conventional features and RFs extracted from [^18^F]F-fluoro-phenylalanine (FBPA) PET/CT and PET/MR obtained from the same cohort of patients, and further developed equations that enable the interchangeability of RFs between PET/CT and PET/MR images with satisfactory comparability. In terms of conventional features, a significant comparability was observed between PET/CT and PET/MR, with 81.2%, 37.5%, 51.5%, and 25% for histograms, geometry, texture, and wavelet-based features, respectively. In order to enable the exchangeability of quantitative features between PET/CT and PET/MR, linear regression (LR) models were developed for features that exhibited high and moderate consensus. With the application of the proposed equations, it has been demonstrated that conventional features as well as 71% of RFs, particularly those based on histogram and texture analyses, allow for interchangeability between [^18^F]F-FBPA PET and diverse hybrid imaging modalities. The findings of this study may potentially enhance the feasibility of future investigations aimed at comparing PET/CT and PET/MR modalities.

### 2.4 ∣ Model building and validation

ML algorithms utilize as input the image features extracted from radiomics or clinical data as input and generate output labels, which can encompass the disease diagnosis, patient condition, therapeutic outcome, among others,^[68]^ to produce more precise predictive models in lieu of traditional statistical techniques.^[45,83-87]^ The application of ML algorithms, including but not limited to random forest (RF), support vector machine (SVM), Gaussian naive Bayes (GNB), artificial neural network (ANN), KNN, adaptive boosting (AdaBoost), logistic regression , *K*-means clustering, principal component analysis (PCA), and hierarchical clustering, has demonstrated potential to enhance both predictive and diagnostic accuracy.^[87-90]^

The RF algorithm generates an ensemble of decision trees, collectively known as a forest, which can effectively tackle classification or regression tasks. Additionally, an internal optimization technique is utilized to determine an importance score for each feature, reflecting their respective contributions to the optimization process.^[68]^ SVM is a supervised algorithm commonly used for binary classification or regression. Through the utilization of the SVM classifier, the influence of individual training samples can be projected back to the original feature space, which enables the computation of a score reflecting the significance of each feature.^[91]^ The GNB classification model conforms to a Gaussian probability distribution and postulates that the features exhibit independence among themselves. The simple computation units of the ANN process the input data and convey the results to the next layer of neurons (inspired by biology). It is common for neurons to calculate their output by weighting inputs and biasing outputs, followed by nonlinear transformations (e.g., thresholding). The KNN method is among the most popular non-parametric regression methods, based on the assumption that predictor values are distributed non-parametrically according to a kernel function distribution. The AdaBoost algorithm is a method for enhancing the efficacy of weak classifiers in the classification of data. This approach involves iteratively training weak classifiers while assigning varying weights to training samples to increase the accuracy of classification outcomes. By employing this process, AdaBoost aims to improve the overall performance of classification models by increasing the number of correctly classified instances. Logistic Regression datasets consist of *n*-dimensional continuous features, and the resulting model is an *n*-dimensional hyperplane (hence the linearity) that best fits the given dataset. In *K*-means clustering, clusters are referred to as sets of data points that are close to one another, and data points are assigned to clusters based on their closest similarity to create *K* data clusters. PCA is an unsupervised algorithm used to simplify data by constructing linear combinations of building blocks (e.g., features). PCA is an unsupervised algorithm utilized for the purpose of data simplification. This is achieved through the construction of linear combinations of fundamental building blocks, such as features. Hierarchical clustering is a method for identifying similarities within a dataset by iteratively grouping the closest neighboring data points into pairs and clusters, until all the available data points have been assigned to a cluster. Understanding the principles of ML algorithms will enable nuclear medicine professionals to better integrate them into research and clinical practice. The foregoing exposition provides a succinct overview of the ML algorithms discussed herein. A detailed discussion of these algorithms, however, it is beyond the scope of this paper and may be consulted in the recent review authored by Currie and Rohren.^[92]^

The performance of ML algorithms depends on numerous factors, encompassing but not limited to data size and randomness during learning or pre-processing steps.^[93]^ Training and validation are required for the generated models. In this process, the error rate is defined and an estimate is calculated that is the closest to the true data.^[84,94]^ Verification methods include the hold-out approach and cross-validation strategies.^[68]^ The validation process can be conducted either internally or externally.^[45]^ Internal validation is susceptible to potential selection bias, whereas external validation employs a separate dataset for validation, thereby offering greater generalizability.^[84]^ It is necessary to use external validation and confirm observations in multicenter settings to further clinical translation.^[95]^ It is possible to evaluate accurate models based on the area under the curve (AUC).^[96]^ AUC scores closer to 1.0 indicate a stronger discriminatory power.

## 3 ∣ RADIOMICS APPLICATION IN DIGESTIVE SYSTEM TUMORS

Solid tumors exhibit both spatial and temporal heterogeneity across multiple levels, including genetic, cellular, protein, microenvironmental, tissue, and organ levels. This complexity poses significant challenges for the accurate and representative characterization of tumors through tissue biopsy alone.^[18,71,97]^ Radiomics enables the capture of tumor heterogeneity throughout the entire volume, in a manner that is non-invasive, expeditious, and straightforward to execute.^[66,98]^

### 3.1 ∣ Esophageal/gastroesophageal cancer

A yearly global incidence of 572,034 new cases of esophageal/gastroesophageal (GOJ) cancer has been estimated.^[19]^ It continues to be a key challenge to stratify patients for treatment in GOJ patients, as a considerable number of patients experience poor quality of life after surgery.^[99]^ Baiocco et al.^[19]^ aimed to investigate the potential association between [^18^F]FDG PET/MR RFs and metastases in the staging process of patients diagnosed with GOJ cancer. The study cohort ultimately comprised 20 patients, of whom 10 had developed metastases. Combining lower SUV second-order (GLCM)( entropy with higher apparent diffusion coefficient (ADC) GLCM entropy, representing lower and higher local voxel heterogeneity (or irregularity), respectively. This approach yielded a sensitivity and specificity of 80% in detecting the presence of metastatic disease, with statistical significance (*p* < .001). The quantitative combination of [^18^F]FDG PET and MRI RFs derived from the primary tumor in simultaneous PET/MR scans exhibited an association with a metastatic phenotype, potentially aiding in the identification of patients who may require alternative therapeutic strategies or closer surveillance in the future.

At present, the conventional method for managing locally advanced resectable thoracic EC involves administering neoadjuvant chemoradiotherapy (NACRT) followed by esophagectomy.^[100]^ Clinical studies have indicated that the rates of pathological complete response (pCR) resulting from NACRT fall within the range of 25%–50%. However, a subset of patients, approximately 15%–20%, do not display any observable response to the treatment.^[100-102]^ The development of dependable predictive biomarkers has the potential to enhance treatment optimization by facilitating the precise selection of patients. This strategy could entail the administration of dose-intensified NACRT to individuals who are potential non-responders, while also taking into account the potential for organ preservation by avoiding esophagectomy or reducing RT dosage for those expected to have a complete response.^[101,103-105]^ [^18^F]FDG PET has been a longstanding imaging technique used to stage EC prior to treatment and re-stage the disease following neoadjuvant therapy by detecting changes in cancer metabolism.^[106-109]^ Rishi et al.^[28]^ used combined pretreatment CT and PET/CT to develop and validate a radiomics-based model for predicting the rate of pCR in patients with locally advanced EC following NACRT. Furthermore, the authors investigated the potential of this model to predict loco-regional control (LRC). There was a significant relationship between tumor regression grade (TRG) 0–1 indicating complete remission or minimal residual disease and improved 5-year LRC (*p* = .020). In CT, PET, and CT + PET predictive models, an improvement in predicting power was achieved with the composite model, with an AUC of 0.87 ± 0.06. Stratifying patients based on their radiomic scores (Rad-scores), the low-score group showed clinically meaningful improvement over the 5-year LRC (*p* = .2). The objective of this investigation is to incorporate radiomics-guided radiotherapy into a routine patient-specific treatment paradigm based on encouraging results. Xiong et al.^[29]^ developed an RF model that involved both clinical and discriminative RFs, and the findings revealed a satisfactory level of accuracy in predicting the local control status of patients with EC who received chemoradiotherapy (CRT). It may be possible to enhance the predictive accuracy of information by leveraging RFs that are derived from high-frequency decompositions, specifically wavelet features, as well as those that are collected across multiple pretreatment time points. The analysis revealed 25 distinctive RFs, consisting of 14 mid-CRT features (AUC_mean_ = 0.746) and 11 pre-CRT features (AUC_mean_ = 0.746), suggesting that mid-CRT SUV images may provide superior information for predicting local control as compared to pre-CRT SUV images. Some RFs as quantitative image biomarkers require further optimization and improvement before they can be routinely implemented in clinical practice. To enhance the efficacy of radiomics-based models in predicting complete response to NACRT among patients diagnosed with EC, Beukinga et al.^[30]^ successfully integrated HER2 and CD44 into the radiomic models, leading to an enhancement of the overall performance and improved discrimination AUCs of 0.759, 0.857, and 0.816, respectively. There was good to moderate calibration in all models. Anconina et al.^[27]^ demonstrated that incorporating RFs derived from hybrid PET/CT, along with sarcopenia measurements from pretreatment [^18^F]FDG PET, can enhance outcome prediction in surgically treated patients with esophagogastric adenocarcinoma. The addition of sarcopenic status (*p* = .051), CT features (*p* = .042), and PET features (*p* = .011) significantly improved the model fit for relapse-free survival.

Chen et al.^[31]^ conducted a study with the aim of exploring the potential of integrating traditional parameters and [^18^F]FDG PET RFs for evaluating treatment response and predicting survival in patients diagnosed with esophageal squamous cell carcinoma (SqCC). In this study, the authors developed a scoring system using independent survival prognosticators, which was then compared to the conventional tumor node metastasis (TNM) staging system and surgical pathology. The findings of the study suggest that a histogram entropy ≥3.69 is a negative prognostic factor for incomplete pathological remission following NCCRT (odds ratio = 19.25, *p* = .009). Furthermore, SUV_max_ reduction ratio ≤ 0.76 and RF code similarity ≤0.0235 of [^18^F]FDG PET imaging serve as early predictive biomarkers for survival following definitive treatment. Prognostic stratification in patients with NCCRT and surgery can be enhanced by combining traditional parameters and RFs with clinical profiles, which could potentially optimize individualized treatment strategies. Patients with esophageal SqCC treated using the tri-modality method can be predicted by the [^18^F]FDG PET-derived radiomic parameter. By integrating traditional and radiomic PET parameters with clinical profiles facilitates, it becomes possible to better stratify patients into distinct subgroups with varying survival rates.

### 3.2 ∣ Gastric cancer

Gastric cancer (GC) is the fifth most prevalent cancer worldwide and represents the third leading cause of cancer-related deaths globally.^[110]^ HER-2 expression status has emerged as a crucial indicator for GC patients, guiding targeted therapy decisions, and accurate assessment of HER-2 gene status at diagnosis plays a pivotal role in clinical treatment planning.^[111,112]^ Liu et al.^[20]^ demonstrated the superior diagnostic performance of [^18^F]FDG PET/CT radiomics in distinguishing HER2+ expression from HER2− expression and predicting prognosis in patients with GC. The ML models, developed using a balanced bagging approach, successfully differentiated HER2+ expression from HER2− expression in the independent validation cohort (IVC) with an AUC of 0.72, sensitivity of 0.85, and specificity of 0.80. The IVC analysis for prognosis prediction yielded an AUC of 0.75, sensitivity of 0.82, and specificity of 0.71.

Lymph vascular invasion (LVI) is an unfavorable prognostic indicator in GC and is known to contribute to local recurrence and distant metastasis of tumors.^[113-115]^ Accurate preoperative identification of LVI status remains challenging since LVI is primarily detected through postoperative pathology. Yang et al.^[21]^ proposed that PET/CT-based radiomics analysis could serve as a non-invasive approach to predict LVI status in GC patients and provide effective predictors of patient survival outcomes. Receiver operating characteristic (ROC) analysis demonstrated the clinical utility of PET/CT RFs combined with clinical data, with AUC values of 0.936 and 0.914 for training and validation, respectively. Moreover, PET/CT RFs alone showed superior performance with AUC values of 0.881 and 0.854 for training and validation, respectively, compared to CT RFs (0.838 and 0.824) and PET RFs (0.821 and 0.812). The PET/CT RF model incorporating tumor grade and SUVmax exhibited excellent clinical value, suggesting the additional value of clinicopathological variables and metabolic parameters in the identification of LVI status in GC patients. Fan et al.^[22]^ investigated the value of PET/CT radiomics and clinical variables in predicting LVI status in GC before surgery. Ten RFs and eight clinical factors were selected to develop predictive models. The combined models demonstrated superior performance compared to both the image models and the clinical models, with AdaBoost, linear discriminant analysis (LDA), and logistic regression classifiers achieving AUC values of 0.944, 0.929, and 0.921, respectively. The study also compared the performance of three ML classifiers—AdaBoost, LDA, and logistic regression—and found no significant difference.

Clinicians currently use the TNM staging system for risk stratification and treatment management of GC, but this does not provide full prognostic information. Lymph node metastasis (LNM) is the most frequently observed pathway of gastric GC metastasis, significantly impacting patient prognosis and influencing the development of individualized treatment strategies.^[116,117]^ However, conventional [^18^F]FDG PET/CT diagnosis is relatively insensitive to detecting LNM in GC due to low spatial resolution and the presence of lesions lacking obvious [^18^F]FDG uptake.^[118,119]^ Xue et al.^[32]^ developed a radiomics nomogram that offers supplementary information for the personalized preoperative prediction of LNM in GC patients. The investigators included clinical data together with conventional imaging features in a multifactorial regression analysis, and finally established a nomogram containing three features of PET/CT diagnosis of LNM, carbohydrate antigen (CA) 19-9 and PET/CT Rad-score, and validated it in two centers, with the final results predicting LNM with AUCs of 0.861, 0.889, and 0.897, which has high diagnostic value. Radiomics nomogram robustness will need to be verified in future multicenter and multidisciplinary studies. Liu et al.^[23]^ developed and validated predictive ML models based on [^18^F]FDG PET/CT radiomics to predict LNM and specific N stage in GC. The radiomics model using [^18^F]FDG PET/CT demonstrated superior performance in discriminating LNMs, achieving an AUC of 82.2% in the independent validation set. Furthermore, it successfully detected LNMs that were missed by contrast-enhanced computed tomography (19.6%) and [^18^F]FDG PET/CT (35.7%), highlighting the potential of the PET/CT-based radiomics model to complement [^18^F]FDG PET/CT and optimize diagnostic accuracy. Peritoneal metastasis (PM) is the most frequent form of metastasis among G) patients.^[120,121]^ It is also the primary factor contributing to decreased survival time in GC patients.^[122]^ Xue et al.^[24]^ assessed the value of a computer-assisted method utilizing a large volume of clinical and PET data to predict PM preoperatively in GC patients. The study demonstrated that the comprehensive model, incorporating Rad-score, SUV_max_, and CA125, exhibited promising predictive value for PM in GC patients, with an AUC of 0.90 in the training cohort and 0.88 in the validation cohort.

Due to the heterogeneity of tumors, clinical outcomes can differ significantly even among patients with similar stages and treatments. Jiang et al.^[33]^ conducted a study in which quantitative RFs were identified from [^18^F]FDG PET to differentiate patients into low- and high-Rad-score groups. These groups exhibited significant discrepancies in 5-year overall survival(OS) and disease-free survival (DFS), as well as predicted the survival and chemotherapy benefit in GC patients. Multivariate analysis revealed that the Rad-score is an independent prognostic factor. Radiomics-based nomograms that included Rad-scores (*C*-index: DFS, 0.800; OS, 0.786) performed better than clini-copathologic nomograms and TNM staging, highlighting the efficacy of radiomic signatures in accurately estimating DFS and OS. According to further analysis, patients with higher Rad-scores had a better chance of benefiting from chemotherapy. In addition, future studies should assess the relationship between imaging signatures and genomic sequencing. The tumor immune microenvironment plays a crucial role in cancer progression, metastasis, and therapeutic response.^[123,124]^ Li et al.^[34]^ developed a radiomics signature of tumor immune microenvironment score (RTIMS) based on PET/CT images to evaluate its predictive ability for survival and the benefits of adjuvant chemotherapy. The study revealed that adjuvant chemotherapy provided a significant survival benefit to patients with stage II and III GC classified as high-RTIMS (for DFS, stage II: Hazard Ratio(HR) 0.208, *p* = .012; stage III: HR 0.321, *p* < .001), while low-RTIMS patients did not show significant benefits from adjuvant chemotherapy. However, it should be noted that the decision to undergo postoperative chemotherapy was made collaboratively by patients and clinicians and was not assigned randomly, which represents a limitation of this study.

### 3.3 ∣ Primary gastric lymphoma

Primary gastric lymphoma (PGL) is an uncommon neoplasm, with DLBCL accounting for 59% of cases.^[125]^ The findings of Zhou et al.^[26]^ indicate that various texture parameters derived from [^18^F]FDG PET and low-dose CT can have the potential to predict the clinical outcome of patients with PG-DLBCL, thereby facilitating the adoption of personalized treatment approaches. Multivariate analysis identified kurtosis (*p* = .012), metabolic tumor volume (MTV) (*p* = .011), and gray-level nonuniformity (GLNU) (*p* = .002) as independent prognostic factors in PET imaging, while sphericity (*p* = .025), kurtosis (*p* = .016), GLNU (*p* = .042), and high gray-level zone emphasis (HGZE) (*p* = .024) were identified as independent prognostic factors in CT imaging. The clinical markers and metabolic baselines assessed through [^18^F]FDG PET/CT parameters did not demonstrate any significant differences in terms of survival, most likely because the study population was small.

### 3.4 ∣ Hepatobiliary tumors

Hepatocellular carcinoma (HCC) is the most frequently occurring type of primary liver cancer and is the second major contributor to cancer-related deaths globally.^[110]^ Intrahepatic cholangiocarcinoma (IHC) is the second most common liver malignancy, and is characterized by aggressive biology leading to a poor response to systemic therapies and an unfavorable prognosis.^[126,127]^ RFs of [^18^F]FDG PET/CT that incorporate clinical factors can be useful for the classification of HCC and IHC, as well as the prediction of microvascular invasion (MVI) based on [^18^F]FDG PET/CT. Jiang et al.^[10]^ explored the potential applications of an ML model with RFs and clinical factors in HCC and IHC classification and detection of MVI presence before surgery. The predictive ability of PET features is prominent in the performance of the model. For HCC and IHC classification, radiomics predictors composed of two PET (Log-sigma-3-0-mm-3D_shortrunhighgraylevelemphasis_PET and Wavelet-HHL_Variance_PET) and one CT (Wavelet-LHL_Median_CT) features achieved an AUC of 0.86 in the test cohort. Regarding the prediction of MVI, three PET features (Log-sigma-3-0-mm-3D_Range_PET, Wavelet-HHH_totalenergy_PET, and Wavelet-LLH_Entropy_PET) along with tumor stage features have exhibited substantial predictive power in the HCC group (AUC = 0.88). In contrast, two PET features (Wavelet-HLL_Minimum_PET and Wavelet-HLL totalenergy_PET) and CA19-9 have demonstrated strong predictive ability in the IHC group (AUC = 0.90). Prediction models provide non-invasive biomarkers for early diagnosis and comprehensive quantification of liver cancers. Fiz et al.^[36]^ investigated the predictive and prognostic roles of PET-based radiomics in patients with surgically treated mass-forming IHC, analyzing the RFs of both the tumor and the peritumoral tissue. The models combining clinical data and RFs of the tumor-VOI showed superior performance compared to clinical models (AUC = 0.78 vs. 0.72 for grading; 0.87 vs. 0.78 for MVI). The inclusion of RFs extracted from the margin-VOI further improved the prediction of grading (AUC = 0.83), although not for MVI. While the study demonstrated the optimization of tumor grading and survival prediction by incorporating textural features from the margin-VOI, additional analyses are warranted to elucidate the association between peritumoral radiomics and pathology data.

Numerous studies have been conducted to identify precise predictive factors for guiding therapy, and several robust predictors of mortality have been identified.^[128-131]^ Blanc-Durand et al.^[35]^ utilized a pretreatment [^18^F]FDG PET-based radiomics signature of the whole liver to develop a PET radiomics scoring system for predicting both PFS and OS in patients with unresectable HCC undergoing transarterial radioembolization using yttrium-90 (^90^Y-TARE). The main RFs that contributed to the scoring system were strength and variation, which were able to effectively stratify patients into low- and high-risk groups for both PFS and OS. Furthermore, this scoring system retained its statistical significance (*p* = .001) in multivariate analysis, independent of the Barcelona Clinic Liver Cancer staging system.^[131]^

### 3.5 ∣ Pancreatic cancer

**FIGURE 3:**
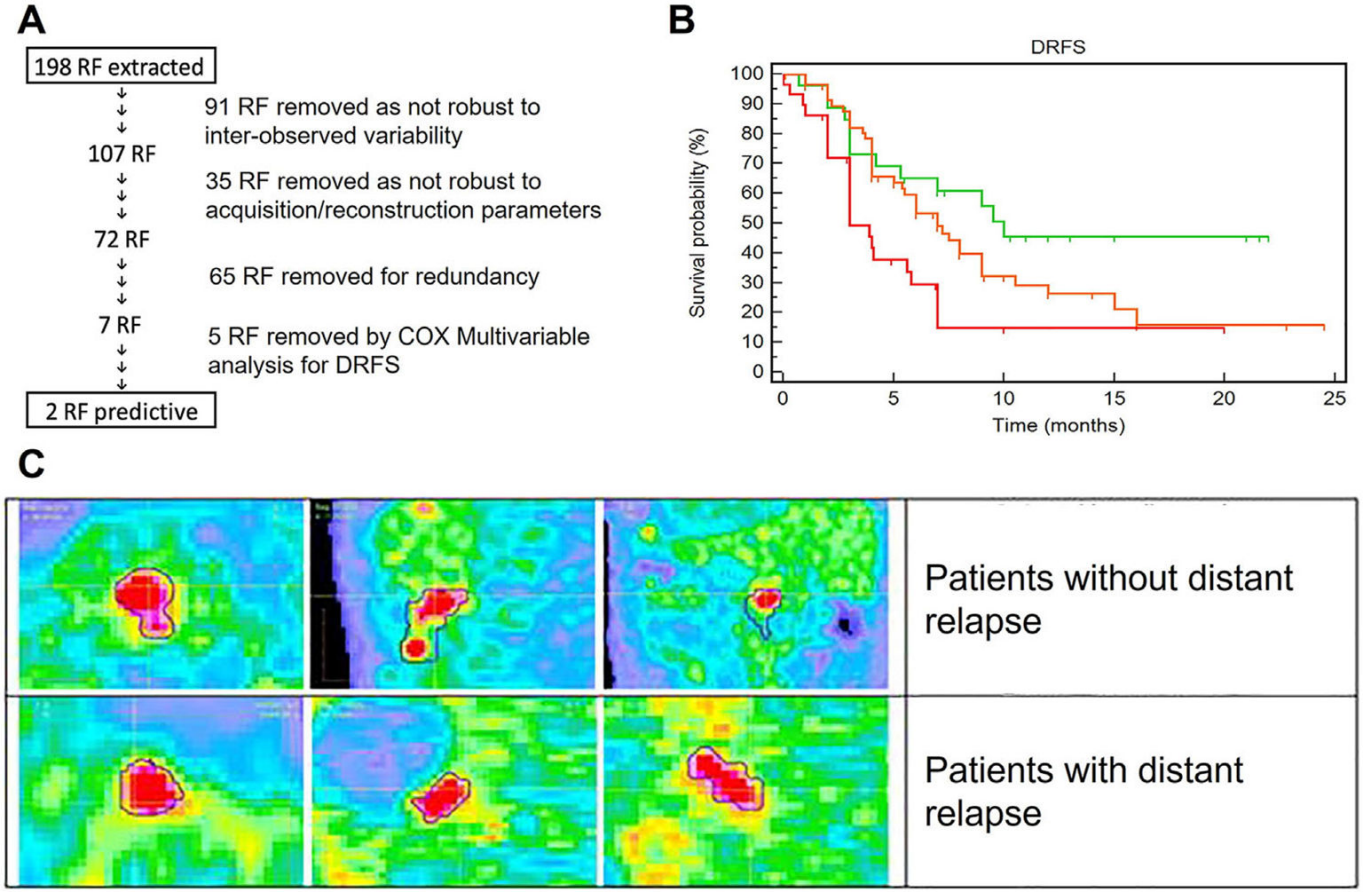
Analysis of a positron emission tomography (PET) random forest (RF) model in predicting distant relapse-free survival in patients after radio-chemotherapy for locally advanced pancreatic cancer. Plot of the RF selection procedure (A). Distant relapse-free survival probability curves according to P_INDEX values stratification for training cohort (B). In the upper part, the lesion uptake is not distributed uniformly in the region of interest (ROI), and the patient had no distant relapse at death. In the lower part, the lesion uptake is highly concentrated and uniformly distributed in the ROI, and early distant metastases were reported in this patient (C). Reproduced under the terms of the 5571690652510 license.^[37]^ Copyright 2020, Copyright Mori et al.

Pancreatic cancer has a notably poor prognosis, with only 9% of patients having a 5-year OS rate.^[132]^ Patients with pancreatic cancer can benefit from [^18^F]FDG PET as a diagnostic imaging technique that detects metastatic lesions and predicts clinical outcomes.^[133,134]^ The degree of inflammatory response to cancer cells can be inferred by assessing FDG uptake in the bone marrow (BM), which has been found to be significantly associated with survival in various cancer types.^[135-138]^ In 65 patients with pancreatic cancer, Lee et al.^[139]^ evaluated the prognostic significance of FDG uptake of BM for predicting OS and demonstrated that integrated FDG uptake of BM and RFs could further improve prognostic stratification based on the “seed and soil theory.”^[137,140,141]^ An important finding was the significant positive correlation observed between BM imaging parameters and serum inflammatory markers. Multivariable analysis revealed that BM SUV, total lesion glycolysis (TLG), and first-order entropy of pancreatic cancer were significant independent prognostic indicators of OS. The integration of PET/CT parameters of primary tumors and BM into a scoring system can yield prognostic information that is independent of tumor stage and treatment. Based on subgroup analysis, the scoring system offered robust prognostic stratification regardless of tumor stage or treatment modality. However, since the study was retrospective, additional factors reflecting the host immune status, such as interleukin 6, transforming growth factor-beta, and BM biopsy findings, could not be assessed. There is a need for further investigation using histopathological and laboratory analyses to elucidate the underlying mechanisms linking FDG uptake in the BM and prognosis in patients with pancreatic cancer. To intensify local and/or systemic therapies for patients with favorable prognosis while avoiding overtreatment in those with poor expected outcomes, Mori et al.^[37]^ assessed the prognostic significance of [^18^F]FDG PET-based RFs for predicting distant relapse-free survival (DRFS) in patients with locally advanced pancreatic cancer (LAPC) who received radiochemotherapy. The results showed that robust PET-based RFs such as Center of Mass Shift (COMshift) and 10th intensity percentile (P10) were strong predictors of DRFS after CRT, with a significantly higher ability to stratify risk categories than currently available biomarkers or clinical features (*p* = .0005, HR = 2.72). In addition, a multicenter independent validation involving three different institutes is underway (Figure 3).

### 3.6 ∣ Neuroendocrine neoplasms

Radiomics was recently applied to manage NETs, extremely rare tumors.^[70]^ The evaluation of tumor grade typically involves invasive techniques such as EUS FNA/FNB. Bevilacqua et al.^[25]^ used [^68^Ga]Ga-DOTANOC PET/CT-derived RFs to predict grade in G2 and G1 primary panNET patients. They concluded that the optimal approach is to use a model trained solely on patients with histological ground truth (test AUC = 0.90), with the most effective RFs being the couple made of GLCM normalized homogeneity and entropy, which exhibited strong robustness to volume segmentation variations.

### 3.7 ∣ Colorectal cancer

**FIGURE 4:**
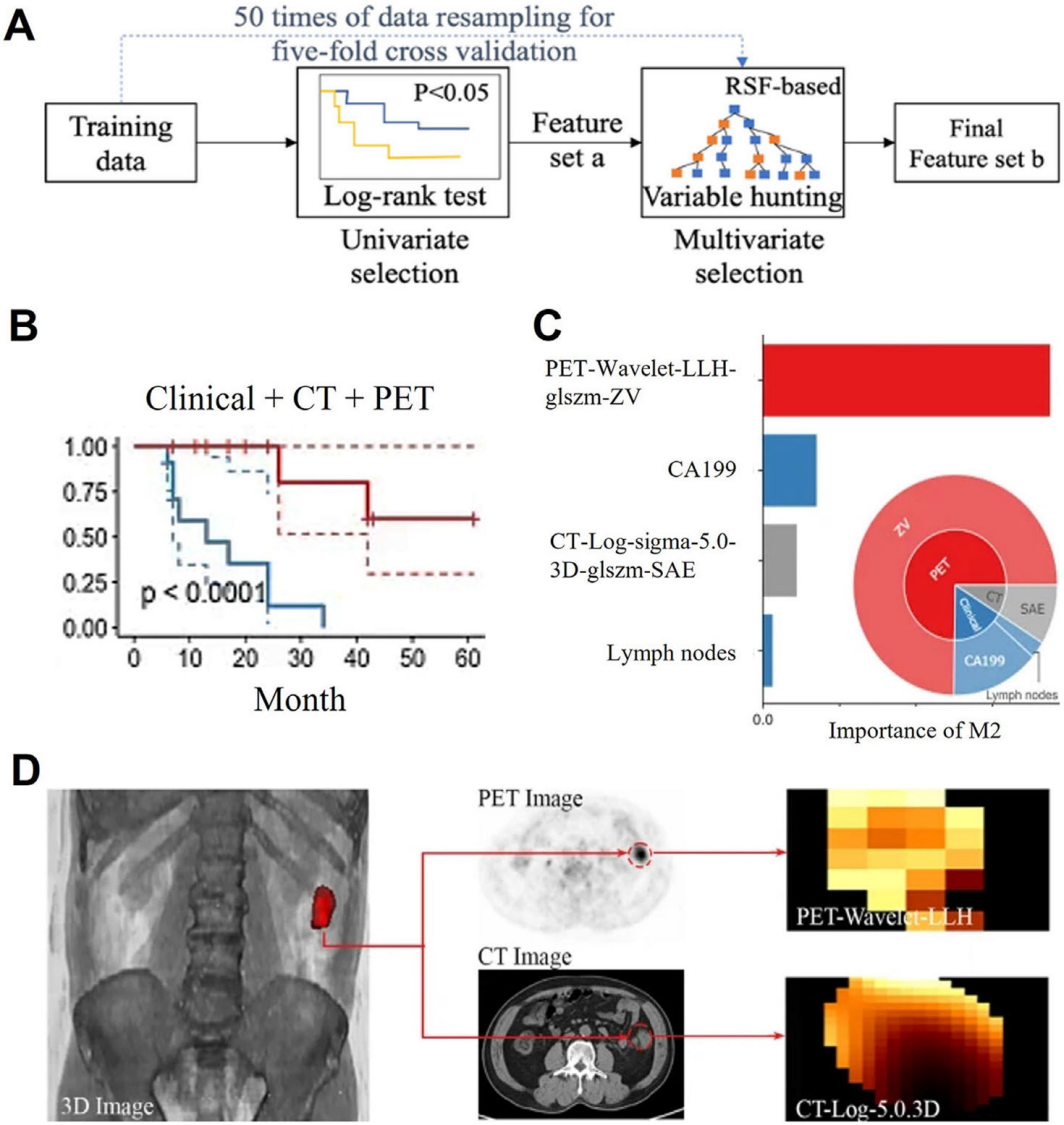
Radiomic analysis for predicting prognosis of colorectal cancer in positron emission tomography (PET)/computed tomography (CT). Methodology of radiomics feature selection (A). Kaplan–Meier (K-M) curves for clinical + CT + PET model on stage III patients (B). The univariate importance of features in the model on stage III patients (C). The visualization of the tumor region in 3D body imaging, in PET/CT imaging and PET/CT radiomics features (D). Reproduced with permission.^[39]^ Copyright 2022, Springer Nature.

Across the globe, colorectal cancer (CRC) is a common cancer that is often associated with liver metastases.^[142]^ Approximately 15% of patients with CRC present with liver metastases at the time of diagnosis, and an additional 15% of patients develop liver metastases subsequently.^[143]^ By incorporating PET-derived RFs, Rahmim et al.^[38]^ developed prognostic models to assess which methods are most effective for predicting clinical outcomes in 52 patients with liver metastases from CRC. The results demonstrated that commonly invoked SUV metrics showed poor performance. For OS, PFS, and EFS, SUV_max_ HRs were 1.48, 0.83, and 1.16, while SUV_peak_ HRs were 2.05, 1.93, and 1.64, respectively. In contrast, the liver metastasis count, TLG, and MTV demonstrated commendable performance. The enhanced prediction was obtained by incorporating multiple features (including those quantifying intra-tumor heterogeneity) into multivariate prognostic models for OS, PFS, and event-free survival (EFS), and HRs of 4.29, 4.02, and 3.20 were obtained (*p*-values of .00004, .0019, and .0002). There is potential for significant predictors of outcomes in patients with colorectal liver metastases based on volumetric and/or heterogeneity features. The improved ability to predict clinical outcomes can facilitate non-invasive selection of patients for personalized treatment or participation in clinical trials involving diverse therapeutic regimens. Based on clinical–biological and [^18^F]FDG PET/CT-based RFs, Lv et al.^[39]^ developed a survival model via ML to predict the prognosis of CRC (Figure 4). The radiomics signatures, comprising four PET/CT features and four clinical factors, demonstrated the most effective prognostic prediction among 196 patients, with a *C*-index of 0.780. It is noteworthy that patients with stage III CRC constitute approximately one-third of all patients, as per the data compiled by the National Cancer Data Base (NCDB). Among this largest proportion of patients, the 5-year survival rate was highly variable. Therefore, the study applied the same methodology to examine the variations among 78 stage III CRC patients. Radiomics signatures (including two clinical features and two RFs) were selected for prognostic prediction (*C*-index = 0.820). The Kaplan–Meier survival curves for both models exhibited a substantial stratification of low- and high-risk cohorts (*p* < .0001). There was a correlation between selected RFs and tumor metabolic factors, such as SUV_mean_ and SUV_max_, based on Pearson correlation analysis. The author intends to undertake prospective studies to validate the model and explore the prognostic implications for patients belonging to sub-stages such as IIIA, IIIB, and IIIC.

### 3.8 ∣ Anal squamous cell carcinoma

Anal squamous cell carcinoma (ASCC) is on the rise globally and is typically treated with CRT for non-metastatic cases.^[144]^ Most cases have loco-regional treatment failure (LRF), and accurate prognostication of PFS would help tailor CRT personalized regimens to each patient.^[145,146]^ There have been some studies evaluating MTV and SUV_max_ as predictors of local recurrence and OS in ASCC patients.^[147-149]^ Brown et al.^[40]^ conducted a study with 189 participants, and found that RFs derived from baseline [^18^F]FDG PET/CT were predictive of the survival outcomes in patients with ASCC undergoing CRT. Researchers have also analyzed the performance of RF prognostic models and conventional feature prognostic models. According to the results, RFs may be more useful in predicting PFS in ASCC than standard features. The RFs of GLCM entropy, NGLDM busyness, the minimum CT value, and a standardized version of MTV may offer improved and more objective prediction of PFS compared to current methods. In the training and validation cohorts, the AUCs for models A (clinical), B (radiomic), and C (radiomic/clinical) were 0.6355, 0.7403, and 0.7142 and 0.6024, 0.6595, and 0.7381, respectively. ASCC patients could benefit from this method as it helps to stratify and personalize their treatment.

## 4 ∣ CHALLENGES AND FUTURE PERSPECTIVES

The adoption of radiomics approach has the potential to serve as the subsequent rational phase in the development of nuclear medicine, which has a well-established history of furnishing quantitative biological information.^[66]^ But in terms of “how to use radiomics in nuclear medicine” as clinical decision support systems, there are challenges and points of high clinical interest to be clarified.^[150]^ Overcoming these challenges and addressing the limitations will be crucial for realizing the full potential of radiomics and translating it into improved patient care and outcomes. In the current review, we discussed these issues and offered recommendations and future perspectives to improve the generalization and clinical utility of nuclear medicine radiomics models.

### 4.1 ∣ Quality control of radiomics data

As a data-driven science, nuclear medicine radiomics research still lacks consensus description in every step from image acquisition to statistical analysis. One major challenge is the lack of standardization in RF extraction, which often leads to variability and inconsistency in results across different studies and imaging platforms. In 2016, the FORCE11 community^[151]^ proposed FAIR scientific data management guidelines, emphasizing the findability, accessibility, interoperability, and reusability of data, which can provide standardized data guarantee for radiomics research.^[152,153]^ Lambin et al.^[67]^ proposed the radiomics quality score (RQS), which can evaluate the quality of both past and future radiomics research. The assessment criteria comprise 16 crucial elements, each of which is allocated a specific number of points that reflects its significance. The optimal radiomics study would attain a cumulative score of 36, indicating fulfillment of all key components.^[154]^ Also, it serves as a reporting set of guidelines for assessing the quality of radiomics studies and verifying whether best practices and procedures were followed by the researchers and reviewers.^[67]^ Following standardized guidelines will help advance applications of radiomics in the future.

### 4.2 ∣ Robustness of features and generalizability of models

The development of prognostic models based on radiomics requires the features to be robust and the models to be generalizable.^[83]^ However, multiple sources of bias can influence radiomic analyses; for example, RFs are sensitive to the variations in scanner model/manufacturer, reconstruction algorithm, acquisition protocols, number of iterations, scan duration, filter after reconstruction, and voxel size.^[155-157]^ In addition to these prerequisites, radiomics studies are frequently hindered by class imbalances and overfitting.^[66]^ If class imbalances exist, the model may still produce high accuracy but with low specificity or low sensitivity, which is not useful in clinical practice. There have been various approaches proposed to address this issue, including augmenting data at the image level and sampling data at the feature level.^[158]^ In overfitting, more features are extracted than samples, resulting in redundant and irrelevant features.^[159]^ Through the utilization of test–retest studies and bootstrapping methods, it is feasible to curtail the number of features by selecting only those that are deemed robust and capable of producing precise results.^[160,161]^

Generalizability of radiomics models is crucial for their clinical application.^[162]^ The manual annotation method makes the reproducibility of some RFs poor, and segmentation by automated and semiautomated methods is becoming more popular because of their rapidity, repeatability, and reproducibility.^[3]^ The location and size of digestive system tumors can pose challenges for radiomic analysis. Tumors located in anatomically complex regions or near adjacent structures can be difficult to segment accurately, leading to potential errors in feature extraction. Advanced segmentation algorithms and techniques specifically designed for challenging tumor locations and sizes can help mitigate these challenges. The results of most early radiomic studies were overoptimistic, and radiomics studies must be reproducible and validated to generate sufficient and convincing evidence.^[45,163]^ In addition, it is necessary to further provide a biological interpretation of RFs.^[97]^ With the increasing pressure for robust radiomics, biological verification will become a standard practice in this field.^[6]^ Additionally, the limited availability of large, well-annotated datasets with longitudinal follow-up data poses a significant challenge for training robust and generalizable radiomic models. We think that increased emphasis ought to be placed on assessing the broader applicability of models and robustness of the RFs through larger, high-quality prospective studies and independent external validation set for multi-institutional collaboration.^[71,95,67,164]^

### 4.3 ∣ Data sharing and management

With regard to publicly sharing cohorts of patients having PET/CT images and pertinent clinical data, such as response to therapy, tumor type classification, and survival, a key issue should be balanced between privacy and regulatory requirements^[7,85]^ In practical terms, these limitations impede the reexamination of identical datasets to support the replication and external validation of established models. The Cancer Imaging Archive (TCIA)^[165]^ and Qualitative Imaging Biomarker Alliance (QIBA)^[166]^ represent a selection of data sharing platforms that are currently available. On the one hand, multicenter radiomics studies must deal with strict regulations governing the ethical and legal aspects of exchanging patient data.^[167,168]^ Conversely, conducting more advanced and comprehensive analyses may necessitate the inclusion of supplementary data (such as survival time and correlated biotest results) that mandate frequent updates to the datasets, thus resulting in substantial expenses associated with data management.^[169]^ As a new model creation method, federated learning uses data collected by different institutions to develop trained consensus models without sharing them and maintaining patient privacy. The computation of data is performed by either aggregation servers or peer-to-peer systems in a decentralized data model. Using this approach, large, heterogeneous, and curated multicentric datasets can be securely accessed for development and evaluation.^[170,171]^ In order to guarantee stringent standardization and dependability throughout all stages of the model development process (from patient recruitment to model assessment), federated learning demands significant investment of resources from participants, particularly with respect to ensuring the generalizability of the models. Finally, compared with negative results, it is easier to publish and report positive results.^[172]^ But negative results should be encouraged to be reported, which would help avoiding repetitive analysis and focus on the relevant models, which may be shifted to the next levels of validation, such as the prospective validation.^[150]^

### 4.4 ∣ ML algorithms

More robust statistical analysis and modeling strategies rely on ML methodology; however, it is becoming increasingly difficult to understand the underlying learning and classification processes as techniques and developed models become more complex.^[173,174]^ Given that ML is fundamentally rooted in statistical principles, it may not be feasible to identify a “deterministic optimal” algorithm or architecture that can yield the most “ideal/optimal” results. Training outcomes are prone to substantial variability based on the initial values of trainable parameters within ML models, as well as slight variations in structure.^[169]^ ML methods are frequently criticized for being black boxes, as their effectiveness and reproducibility lack substantial evidence, owing to the complexity and opacity of the algorithms.^[11,25]^ Furthermore, the interpretability of RFs and the clinical relevance of their associations with outcomes require further investigation to establish their true predictive value. How to “open the black box” and provide interpretable models in clinical practice requires cooperation and efforts among computer scientists, bioinformaticists, statisticians, and nuclear medicine doctors.^[175,176]^

### 4.5 ∣ Development of artificial intelligence

Biomedicine is one of the most important fields for artificial intelligence (AI) applications and has synergistically led to a rapid rise in its potential use in various imaging tasks, which has become increasingly pivotal in biomedical research and clinical practice.^[16,85,162,177,178]^ The integration of radiomics into clinical workflows and the development of user-friendly software tools for radiomic analysis present practical challenges that need to be addressed to facilitate wider adoption of radiomics in routine clinical settings. Thus far, the employment of AI for nuclear medical imaging has demonstrated consistent enhancements. AI is already being used in the technological sector to improve PET image attenuation correction, artifact-free image reconstruction, and anatomical landmarking. The image analysis sector already uses AI to assist in image interpretation, complete automation of disease classification, and fully automated identification of metastases in whole-body scans.^[179]^ The integration of AI into radiomics research aims to realize the long-standing aspiration of developing a prompt and single-step diagnostic “virtual biopsy”; however, whether this goal can be achieved remains uncertain.^[180]^ Future challenges will include capturing radiomic data in the structured reports to enhance patients’ management.

## 5 ∣ CONCLUSIONS

In summary, there has been a substantial amount of research exploring the utility of nuclear medicine radiomics in the context of digestive system tumors. It is a very optimistic and promising field of research that maximizes the diagnostic and prognostic yield of existing images. We anticipate that in the near future, the data acquired from multicenter nuclear medical examinations conducted worldwide will be translated into quantitative radiomics data. This development is expected to accelerate workflow and enhance the precision of diagnosis and decision support prediction capacity, thus establishing a link between nuclear medical imaging and personalized diagnosis and treatment. To translate radiomics into clinical practice, higher publication standards and standardization of workflows are needed.

## Figures and Tables

**TABLE 1:** Summary of studies using positron emission tomography (PET)-based radiomics for digestive system tumors.

Location	Aim	Imaging	Cohort size	Findings	Reference
Diagnosis and differentiation					
Esophageal/gastroesophageal cancer	Association with metastases	[^18^F]FDG PET/MR	*n* = 20	High ADC entropy combined with low SUV entropy was associated with metastases	Baiocco et al.^[19]^
Gastric cancer	Classification of HER2 positive expression and negative expression	[^18^F]FDG PET/CT	*n* = 90	The ML model achieved AUC of 0.72, sensitivity of 0.85, and specificity of 0.80	Liu et al.^[20]^
	Preoperative predictions of lymph vascular invasion	[^18^F]FDG PET/CT	*n* = 148	ROC analysis demonstrated the clinical utility of PET/CT RFs combined with clinical data, with AUC values of 0.936 and 0.914 for training and validation	Yang et al.^[21]^
	Preoperative predictions of lymph vascular invasion	[^18^F]FDG PET/CT	*n* = 101	The combined models showed improved performance than the image models and the clinical models, with the AUC values of AdaBoost, LDA, and logistic regression classifier yielding 0.944, 0.929, and 0.921, respectively	Fan et al.^[22]^
	Predict lymph node metastasis and specific N stage	[^18^F]FDG PET/CT	*n* = 185	Radiomics predictors achieved AUC of 0.822 and 0.737	Liu et al.^[23]^
	Preoperative predictions of peritoneal metastasis	[^18^F]FDG PET/CT	*n* = 355	The composite model achieved AUC of 0.90 and 0.88 in training cohort and validation cohort	Xue et al.^[24]^
Hepatocellular carcinoma	Classification of HCC and IHC	[^18^F]FDG PET/CT	*n* = 127	Radiomics predictors achieved AUC of 0.86	Jiang et al.^[10]^
Pancreatic neuroendocrine tumors	Prediction of grade in G2 and G1	[^68^Ga]Ga-DOTANOC PET/CT	*n* = 58	Radiomics predictors achieved AUC of 0.90	Bevilacqua et al.^[25]^
Prognosis and efficacy evaluation					
Gastric lymphoma	Prediction of the clinical outcome	[^18^F]FDG PET/CT	*n* = 35	Kurtosis, MTV, GLNU in PET and sphericity, kurtosis, GLNU, and HGZE in CT were independent prognostic factors	Zhou et al.^[26]^
Esophagogastric cancer	Prediction of overall survival and relapse-free survival	[^18^F]FDG PET/CT	*n* = 145	RFs integrated with clinicopathological parameters and sarcopenia measurement improve outcome prediction	Anconina et al.^[27]^
Esophageal cancer	Prediction of pCR after NACRT	[^18^F]FDG PET/CT	*n* = 68	The composite model achieved AUC of 0.87	Rishi et al.^[28]^
	Prediction of the local control status treated with CRT	[^18^F]FDG PET/CT	*n* = 30	The RF model achieved an accuracy of 0.933	Xiong et al.^[29]^
	Prediction of complete response to NACRT	[^18^F]FDG PET/CT	*n* = 96	Addition of HER2 and CD44 to the RF models improved AUCs	Beukinga et al.^[30]^
	Prediction of outcomes compared to traditional TNM staging system and surgical pathology	[^18^F]FDG PET/CT	*n* = 44	RFs was superior to TNM system and surgical pathology	Chen et al.^[31]^
Gastric cancer	Prediction of lymph node metastasis	[^18^F]FDG PET/CT	*n* = 224	The prediction mode achieved AUC of 0.897	Xue et al.^[32]^
	Prediction of survival and chemotherapy benefits	[^18^F]FDG PET/CT	*n* = 214	Radiomic score was an independent prognostic factor in multivariate analysis	Jiang et al.^[33]^
	Radiomics tumor immune microenvironment score predicts chemotherapy efficacy	[^18^F]FDG PET/CT	*n* = 230	High RTIMS patients benefitted from adjuvant chemotherapy	Li et al.^[34]^
Hepatocellular carcinoma	Prediction of PFS and OS	[^18^F]FDG PET/CT	*n* = 47	Rad-score appears as an independent predictor for PFS (*p* = .006) and OS (*p* = .001)	Blanc-Durand et al.^[35]^
Intrahepatic cholangiocarcinoma	Prediction of MVI and prognosis	[^18^F]FDG PET/CT	*n* = 74	The combined clinical-radiomic models outperformed the pure preoperative clinical ones and achieved performances non-inferior to the postoperative models	Fiz et al.^[36]^
Pancreatic cancer	Prediction of DRFS	[^18^F]FDG PET/CT	*n* = 176	Two robust PET-RFs predicted DRFS	Mori et al.^[37]^
Colorectal cancer	Prediction of outcome with colorectal liver metastases	[^18^F]FDG PET/CT	*n* = 52	The number of liver metastases, TLG, and MTV performed well in prediction of outcome	Rahmim et al.^[38]^
	Prediction of the prognosis	[^18^F]FDG PET/CT	*n* = 196	Radiomics signature achieved the prognostic prediction well (*C*-index = 0.78)	Lv et al.^[39]^
Anal squamous cell carcinoma	Prediction of PFS	[^18^F]FDG PET/CT	*n* = 199	Radiomic and clinical model achieved AUC of 0.7381	Brown et al.^[40]^

Abbreviations: AUC, area under the curve; CRT, chemoradiotherapy; CT, computed tomography; DRFS, distant relapse-free survival; GLNU, gray-level nonuniformity; HCC, hepatocellular carcinoma; HGZE, high gray-level zone emphasis; LDA, linear discriminant analysis; ML, machine learning; MR, magnetic resonance; MTV, metabolic tumor volume; MVI, microvascular invasion; NACRT, neoadjuvant chemoradiotherapy; pCR, pathological complete response; RF, random forest; ROC, receiver operating characteristic; RTIMS, radiomics signature of tumor immune microenvironment score; TNM, tumor node metastasis.
